# The Law in Institutional Practice

**Published:** 1916-02-19

**Authors:** 


### THE LAW IN INSTITUTIONAL PRACTICE.

### An Important Pronouncement.

The Royal College of Physicians has rendered a notable service both to the public and to the medical profession by its recent action in securing a definite pronouncement from high authorities as to exactly what is and what is not incumbent on a medical practitioner who may be called upon to treat a patient for the results of criminal abortion, indubitable or suspected. Not only have they taken the opinion of a very well-known counsel practising largely in criminal cases; they have also submitted their resolution to the Public Prosecutor. Both these authorities have suggested certain changes in the expression of the views of the College; these have been adopted, and although a judge might not feel bound to take exactly the same views as those thus communicated, we think no practitioner who uses them as his guide can possibly get himself into any serious trouble.

It has long been propounded in text-books of medical jurisprudence that the medical practitioner who is confronted with evidence of accomplished crime has a duty, as every other citizen has, to give information at once to the administrators of the law, the police. There is an equally trite modification of this rule, that facts disclosed by one in the relation of a patient to him are sacred and confidential, and must not be used to the detriment of that person. In other words, a medical man who sees a patient suffering from the after-effects of criminal abortion may not give information of it to the police, because the patient is herself accessory to the crime, and would, therefore, be liable to punishment if her doctor carried out his duty as an ordinary citizen. In practice this latter view has prevailed, and doctors never do inform the police in such cases. In fatal cases they do of course (as a rule) notify the Coroner; and this inevitable duty is not abrogated by what has been said about the seal of professional confidence so long as the patient is alive. So far as we know, no doctor has been called to account in recent years for omitting to inform the police of a case of criminal abortion which has come to his knowledge: tacitly, the police accept the position that he cannot be expected to do so. But the law does not itself confer this immunity upon him; and if any such case were brought before a criminal court, it might go hard with the doctor. Hence the importance of an authoritative guide to conduct such as the Royal College of Physicians has provided.

### A Point for Hospital Residents.

In ordinary practice cases such as those under discussion are rare; or, rather, it is rare to see cases where the criminal origin of the abortion is ascertainable or reasonably suspected. But gynæcologists come across a good many such cases, and hospital residents are not at all uncommonly confronted with them. It is particularly important that such institutional officers as visiting gynæcologists, senior resident medical officers, and house officers in charge of obstetric or gynæcological departments should bear in mind the principles laid down by the Royal College of Physicians, for the sake of their institutions as well as of themselves. It is not necessary to reproduce the substance of the memorandum, for much of it (particularly the sections concerning the taking of depositions from dying patients) recapitulates what is to be found in any medico-legal text-book, and should be familiar to every qualified practitioner. What is important, and to some extent novel, in the resolutions adopted by the College can be briefly stated.

The College is of opinion that a moral obligation rests upon every medical practitioner to respect the confidences of his patient; and that, without her consent, he is not justified in disclosing information obtained in the course of his professional attendance upon her. Also, that every medical practitioner who is convinced that criminal abortion has been practised on his patient should urge her, especially when she is likely to die, to make a statement which may be taken as evidence against the person who has performed the operation, provided always that her chances of recovery are not thereby prejudiced. In the event of her refusal to make such a statement, he is under no legal obligation to take further action, but he should continue to attend the patient to the best of his ability. Before taking any action which may lead to legal proceedings, a medical practitioner will be wise to obtain the best medical and legal advice obtainable, both to ensure that the patient's statement may have value as legal evidence and to safeguard his own interests, since in the present state of the law there is no certainty that he will be protected against subsequent litigation. If the patient should die he ought to refuse to give a certificate of the cause of death, and should communicate with the Coroner. These simple, straightforward rules of conduct have the sanction of the best advice the College has been able to secure, and seem admirable both from the point of view of law and from that of justice and common sense.

